# Minimally Invasive Methods for Staging in Lung Cancer: Systematic Review and Meta-Analysis

**DOI:** 10.1155/2016/1024709

**Published:** 2016-10-13

**Authors:** Gonzalo Labarca, Carlos Aravena, Francisco Ortega, Alex Arenas, Adnan Majid, Erik Folch, Hiren J. Mehta, Michael A. Jantz, Sebastian Fernandez-Bussy

**Affiliations:** ^1^Facultad de Medicina, Universidad San Sebastián, Lientur 1457, Concepción 4080871, Chile; ^2^Division of Internal Medicine, Complejo Asistencial Victor Rios Ruiz, Los Angeles, Chile; ^3^Division of Pulmonary Medicine, School of Medicine, Pontificia Universidad Católica de Chile, Santiago, Chile; ^4^Division of Oncology, School of Medicine, Pontificia Universidad Católica de Chile, Santiago, Chile; ^5^Division of Internal Medicine, School of Medicine, Pontificia Universidad Católica de Chile, Santiago, Chile; ^6^Divisions of Thoracic Surgery and Interventional Pulmonary, Beth Israel Deaconess Medical Center, Harvard Medical School, Boston, MA, USA; ^7^Division of Pulmonary and Critical Care Medicine, Massachusetts General Hospital, Harvard Medical School, Boston, MA, USA; ^8^Division of Pulmonary, Critical Care, and Sleep Medicine, University of Florida, Gainesville, FL, USA; ^9^Unidad de Neumologia Intervencional, Clinica Alemana, Universidad del Desarrollo, Santiago, Chile

## Abstract

*Introduction*. Endobronchial ultrasound (EBUS) is a procedure that provides access to the mediastinal staging; however, EBUS cannot be used to stage all of the nodes in the mediastinum. In these cases, endoscopic ultrasound (EUS) is used for complete staging.* Objective*. To provide a synthesis of the evidence on the diagnostic performance of EBUS + EUS in patients undergoing mediastinal staging.* Methods*. Systematic review and meta-analysis to evaluate the diagnostic yield of EBUS + EUS compared with surgical staging. Two researchers performed the literature search, quality assessments, data extractions, and analyses. We produced a meta-analysis including sensitivity, specificity, and likelihood ratio analysis.* Results*. Twelve primary studies (1515 patients) were included; two were randomized controlled trials (RCTs) and ten were prospective trials. The pooled sensitivity for combined EBUS + EUS was 87% (CI 84–89%) and the specificity was 99% (CI 98–100%). For EBUS + EUS performed with a single bronchoscope group, the sensitivity improved to 88% (CI 83.1–91.4%) and specificity improved to 100% (CI 99-100%).* Conclusion*. EBUS + EUS is a highly accurate and safe procedure. The combined procedure should be considered in selected patients with lymphadenopathy noted at stations that are not traditionally accessible with conventional EBUS.

## 1. Introduction

In recent years, the approach to patients with suspected non-small-cell lung cancer (NSCLC), has changed [1]. Several diagnostic and staging methods have been developed to avoid the use of more invasive techniques [2]. Surgical methods, such as mediastinoscopy, video assisted thoracoscopy (VATS), mediastinal dissection, and lymph node resection, are the reference standard for lung cancer lymph node staging. However, minimally invasive methods, including computed tomography (CT), magnetic resonance imaging (MRI), or positron emission tomography (PET), as well as bronchoscopic methods, are alternatives with low complication rates and these methods are often used as the first approach for confirming or excluding metastatic disease [2, 3]. One of the limitations of radiological studies is the number of false positive and false negative cases; for this reason, tissue samples are needed for cytopathology or histopathology [2–4].

Over the last decade, bronchoscopic modalities such as endobronchial ultrasound with transbronchial needle aspiration (EBUS-TBNA) have emerged as safe methods to obtain tissue from mediastinal or in close proximity to central airways, with an accuracy of 80–90% and an incidence of complications of less than 1% [4, 5]. This minimally invasive approach is limited to certain mediastinal lymph nodes; however, one of the weaknesses of EBUS-TBNA is its inability to obtain tissue from stations 5, 6, 8, and 9 of the IASLC mediastinal lymph node map [6]. In such cases, a complementary approach with endoscopic ultrasound (EUS) is a safe alternative to obtain tissue from all of the mediastinal lymph nodes, except from stations 5 and 6 [4].

Studies of combined EBUS + EUS have included retrospective, prospective, and randomized controlled trials (RCTs). Two systematic reviews and meta-analyses have been published [7, 8] in the past. However, most of the evidence from primary studies has been published in the past five years. The purpose of this systematic review and meta-analysis is to evaluate the utility of EBUS + EUS for NSCLC staging or diagnosis in those patients with suspected NSCLC.

## 2. Materials and Methods

### 2.1. Literature Search and Clinical Eligibility Criteria

Previous descriptions and the protocol for this systematic review and meta-analysis are available in the PROSPERO registry (ID: CRD42015017199) [9]. In this systematic review, two independent reviewers searched the following databases: PubMed (Medline), OVID, Lilacs, Clinical Trials (https://clinicaltrials.gov/), and the Cochrane database. In addition, two metasearches of the TRIP database and Epistemonikos were included up to April 2015 [10]. For maximum sensitivity, meeting abstracts were searched from the European Respiratory Society (ERS; 2008 to 2014), American Thoracic Society (ATS; 2008 to 2014), and American College of Chest Physicians (2008 to 2014).

The search criteria included the following. In the PubMed database, we searched using the following MeSH terms: ((“Carcinoma, Non-Small-Cell Lung” [majr]) AND “Bronchoscopy” [majr]) AND “Ultrasonography” [MeSH]. The search also included the following non-MeSH terms: “endobronchial”, “endobronchial ultrasound” (EBUS) alone or in combination with “non-small cell lung carcinoma”, and “neoplasm staging”. The literature search and inclusion criteria were in accordance with the Cochrane handbook and this systematic review was performed in accordance with the PRISMA statement [11].

The inclusion criteria for this systematic review and meta-analysis were the following: (1) patients older than 18 years; (2) confirmed lung cancer with an indication for mediastinal staging (based on enlarged and/or PET positive lymph nodes); (3) available index test defined as EBUS + EUS with different endoscopes or with the same bronchoscope (EBUS + EUS-B-FNA) and reference standard (surgical methods or clinical followup); and (4) two-by-two diagnostic yield results of specificity, sensitivity, and positive likelihood ratios (LRs).

Two independent authors (GL and CA) performed the literature search, and disagreements concerning study inclusion were resolved by discussion. The full-text versions of the included studies were retrieved, and a manual cross-reference search of the articles was performed with no language restrictions.

### 2.2. Quality Assessment of the Retrieved Articles

A methodological assessment and quality analysis were performed by two independent reviewers (GL and CA) using the diagnostic test accuracy approach from the Cochrane Handbook for Systematic Reviews [12] and disagreements concerning study inclusion were resolved by discussion.

### 2.3. Outcomes Measured

We included the diagnostic yield results (specificity, sensitivity, and LR) from all of the included articles in this systematic review and meta-analysis, and a secondary analysis of only EBUS + EUS with fine needle aspiration performed with the same bronchoscope (EBUS + EUS-B- FNA) was performed. In addition, we analysed adverse events related to EBUS + EUS.

For this study we defined the following terms: sensitivity = positive endosonography (EBUS + EUS)/true positive via surgical staging + false negative; specificity = negative endosonography (EBUS + EUS)/true negative via surgical staging + false positive. The patients that were positive during endosonography were considered as true positive.

### 2.4. Data Extraction and Analysis

Data extraction was performed by two independent reviewers (GL and FO). Two-by-two tables were generated that included true positives, false positives, true negatives, and false negatives. Primary study descriptions, population descriptions, types of studies, and reported adverse events were also extracted, and a new summary table was created.

The extracted data were imported into Microsoft Excel 2010 (Redmond, WA, USA). For the qualitative and quantitative analyses, we used Cochrane Review Manager (RevMan) software, version 5.3, and a random effects model was used for the quantitative meta-analysis of diagnostic yield. We defined significant heterogeneity as *i* ^2^ > 50% and created a symmetrical summary receiver operatic characteristic curve (SROC); the area under the curve was analysed using MetaDisc, version 1.4. Statistical significance was defined by a *p* value less than 0.05. We created a summary table of the findings based on the GRADE approach using GRADEpro software.

## 3. Results

**Figure 1:**
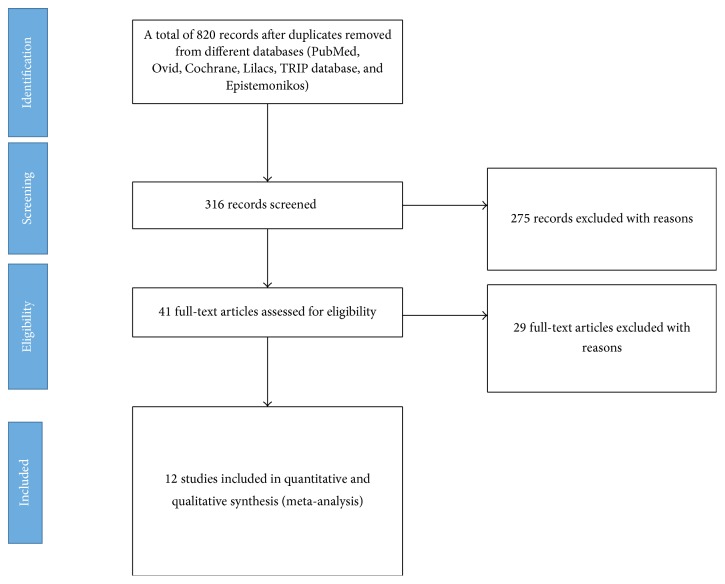
Study of flow diagram following PRISMA statements.

Our search identified 820 records after removing duplicates. 775 references were excluded and a total of 41 potentially eligible primary studies were evaluated in full-text format. After the full-text screening, 29 primary studies were excluded for various reasons [25–54], and 12 primary studies (1515 patients) were included in the qualitative and quantitative analyses and the meta-analysis (Figure 1) [7, 8, 55, 56] [47–54]. No additional studies were identified from conference abstracts. The characteristics of the included and excluded studies are reported in Tables 1 and 5.

Two of the included primary studies were RCTs. Annema et al. allocated patients 1 : 1 to an endoscopic staging arm and a surgical arm. In the other RCT, Kang et al. allocated patients 1 : 1 to an EBUS followed by EUS arm and an EUS followed by EBUS arm. These trials were evaluated by two independent researchers and were defined as the best evidence available.

The remaining studies included in the quantitative and qualitative analyses were observational studies. Eleven of these studies were prospective trials, and the other one trial was retrospective in nature.

### 3.1. Risk of Bias in the Included Reviews

**Figure 2:**
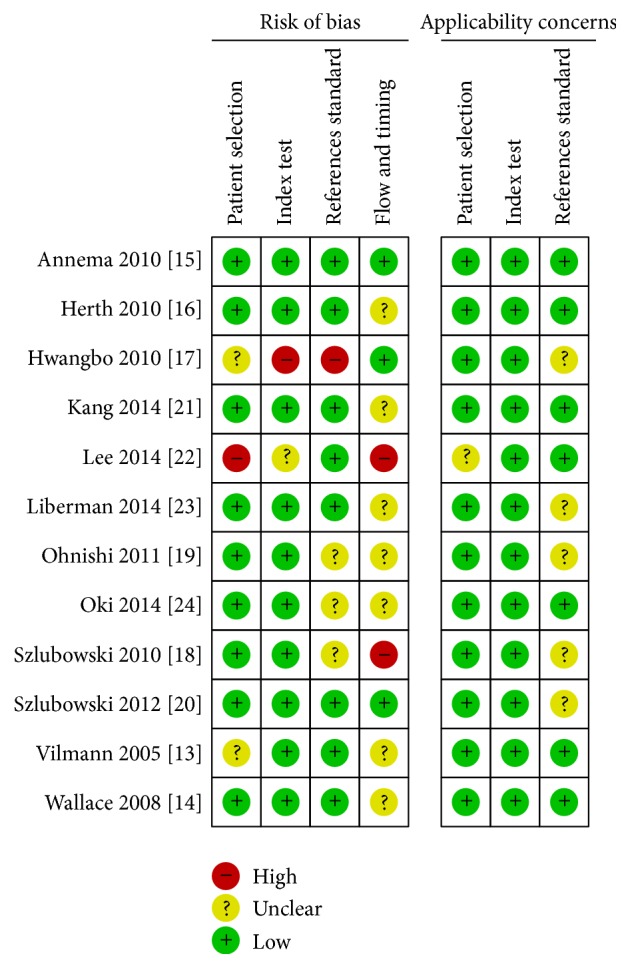
Risk of bias and applicability concerns graph: review of authors' judgments about each domain presented as percentages across included studies.

A quality assessment of the primary studies was performed using the Cochrane assessment tool. Most of the primary studies reported and addressed a specific question (diagnostic yield from EBUS + EUS for mediastinal staging) without any concern for index testing or reference standard. The limitations of the included studies were the following: (1) no data on the interval since the index test or the reference standard; (2) a risk of bias in the results because some patients in the prospective trials were excluded from the data analysis; (3) some tests including “surgical methods” as reference test, without any specification between mediastinoscopy, thoracotomy, and others; and (4) the study type (10 of the 12 trials were prospective). The results are presented in Figures 2 and 5.

### 3.2. Diagnostic Accuracy

**Figure 3:**
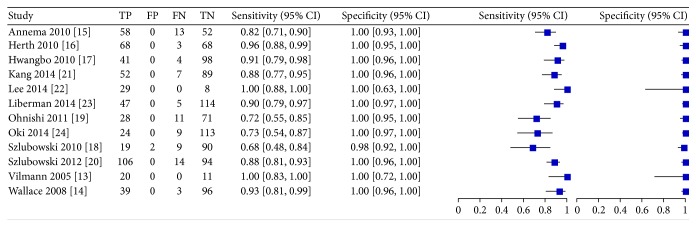
Comparison 1. Forest plot of diagnostic yield from all included studies.

The pooled data from the primary studies that evaluated the diagnostic yield of EBUS + EUS versus surgical methods or clinical followup are shown in Figure 3.

The sensitivity across all the primary studies was 85% (CI 80–89%) and the specificity was 99% (CI 98–100%). The meta-analysis of sensitivity, specificity, and positive likelihood ratio of all the studies and subgroups of EBUS-B-FNA and EBUS + EUS using different endoscopes is reported in Table 3.

**Figure 4:**
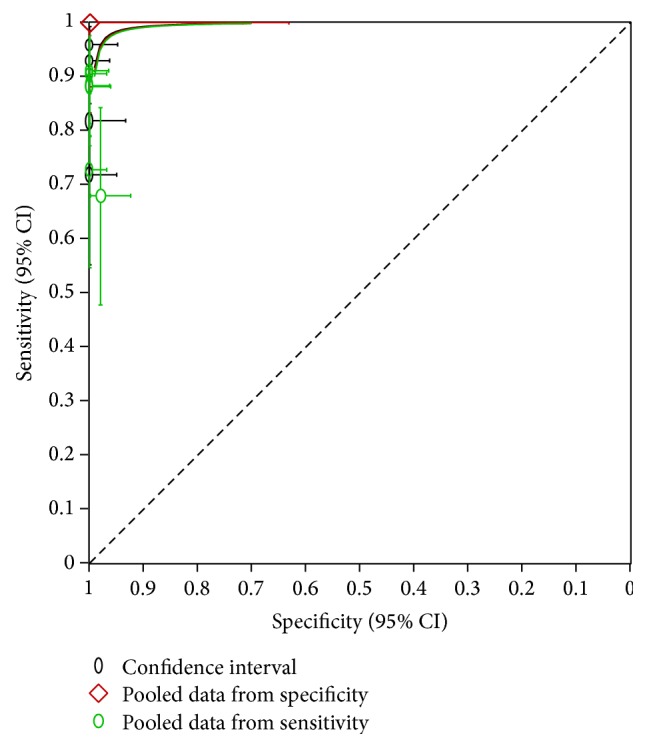
SROC from all included studies.

**Figure 5:**
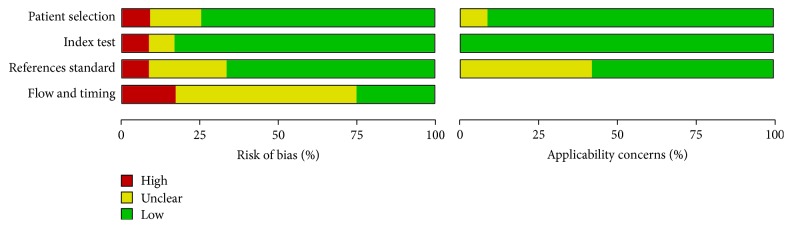
Risk of bias and applicability concerns summary: review of authors' judgments about each domain for each included study.

In a subgroup analysis of the EBUS + EUS using different endoscopes revealed a sensitivity of 85% and a specificity of 99.6%, compared with EBUS-EUS-B-FNA with a sensitivity of 88% and specificity of 100%. Finally, SROC analysis revealed an AUC of 0.98 for all of the included primary studies and 0.99 for the EBUS + ESU-B-FNA only. Summaries of these results are presented in Figure 4.

Adverse events related to endoscopic procedures were reported in 12 primary studies. The most common adverse event was minor bleeding.  Table 2 shows the number of adverse events reported in each trial.

Finally, the quality of the evidence in the EBUS + EUS and EBUS + EUS-B-FNA methods was LOW for sensitivity and MODERATE for specificity based on the GRADE approach. A summary of the findings is provided in Tables 4(a) and 4(b).

## 4. Discussion

EBUS is a minimally invasive procedure with good accuracy in confirming or excluding lung cancer or mediastinal lung cancer metastasis. The addition of EUS to EBUS mediastinal staging, however, has improved the sensitivity and accuracy of this method, thereby decreasing the number of unnecessary thoracotomies and surgical procedures [3, 29, 57]. Based on the published data, we recommend EBUS + EUS as the first step for evaluating patients with suspected operable lung cancer or with known operable lung cancer who require mediastinal staging. In the subgroup analysis of EBUS + EUS-B-FNA data only, the sensitivity improved to 88% and the specificity increased to 100%. In addition, there was a significant decrease in the heterogeneity in the EBUS + EUS-B-FNA only group; these data were consistent with the evidence quality of the primary studies. In a systematic review of surgical mediastinal staging published by Silvestri et al. in 2013, traditional mediastinoscopy had a pooled median sensitivity of 78% and NPV of 91% and video assisted mediastinoscopy had a median sensitivity of 89% and NPV of 92% [57]. EBUS + EUS is a safe procedure. In our review, major adverse related events were reported for less than 1% of the procedures; pneumothorax was the most commonly reported complication, these data confirm the safety of this method across multiple studies in different settings [5].

The evidence quality of the included systematic reviews was assessed using Cochrane tools, which include the most influential aspects of diagnostic test methods (patient selection, index test, reference standard, flow, and timing) [12]. We considered the overall quality of all the included primary studies to be low.

The studies included patients with known lung cancer who required mediastinal staging or lesions suspected to be NSCLC. In most cases, a positive pathologic diagnosis with EBUS + EUS was considered a true positive; in patients with negative diagnoses, a second test was performed (surgical methods or clinical followup). This approach is common in clinical practice, and we considered it to be of little concern to the applicability of our results.

A previous systematic review and meta-analysis was published by Zhang et al. [8]. In that meta-analysis, the risk of bias was evaluated with the STARD and QUADAS tools, and several trials reported low-quality data. In their study, a positive biopsy with an EBUS + EUS procedure was sufficient to confirm the pathological diagnosis, and surgery was not necessary to confirm the disease [58].

According to ACCP guidelines and previous systematic review and meta-analysis, EBUS alone reports a higher diagnostic yield than EBUS/EUS, Dong et al report a sensitivity of 90% (CI, 84.4–95.7%) and a specificity of 98.4% [55, 57]. However, no head to head comparing EBUS, EBUS/EUS, and mediastinoscopy were identified; we found that a network meta-analysis that includes different mediastinal methods for staging in NSCLC is needed.

According to our results, we suggest that EBUS/EUS performed with the same bronchoscope has a higher yield than these performed with separate bronchoscope and endoscope; however, these are not head to head comparisons; so it is difficult to determine what to make of these findings but they are intriguing.

Finally, training is certainly an issue that merits further research. There appears to be a paucity of training in these combined techniques; EUS performed by an interventional pulmonology (not by gastroenterologist trained in EUS) is not taught in most interventional pulmonary fellowships; this fact should be considered as a potential training for interventional pulmonary fellowship programs.

We found some bias in our study. In several studies the reference standards were suboptimal. Mediastinoscopy has an accuracy that is comparable to endosonography. Mediastinoscopy is potentially therefore a suboptimal reference standard and, if used alone, this will lead to overestimations of the accuracy of endosonography. We considered this fact as a major source of bias, and we determined that the protocol of another systematic review was incomplete (PROSPERO ID: CRD42014009792) [56]. We considered this systematic review and meta-analysis as part of the current body of evidence for our study. Limitations of this approach to integrating evidence include the following. First, several nonrandomized studies had different inclusion criteria such as mediastinal masses or lung masses suspected of cancer and included patients with potentially benign lesions. Second, the preprocedure evaluation was not reported in several studies. Some did include the use of PET-CT as part of the preprocedure evaluation. Third, for negative endoscopic procedures, we had concerns about the reference standard. Some patients were excluded from the data analysis in some studies, and in other studies, the final diagnosis was declared using methods other than mediastinoscopy or surgical procedures. All of these various criteria might have introduced heterogeneity in the trials and the RCT data were limited to two trials. More primary studies and RCTs are needed to improve the body of evidence.

## 5. Conclusion

Based on previous primary studies published, this systematic review is the most complete evidence-based synthesis today, including all diagnostic test studies and best quality studies (RCTs) analysed by Cochrane criteria. Based upon this analysis, we believe that EBUS + EUS is a minimally invasive and highly accurate method for mediastinal staging that is associated with a low incidence of complications. While the diagnostic yield is not superior to EBUS alone, the combined procedure should be considered in selected patients with lymphadenopathy noted at stations that are not traditionally accessible with conventional EBUS. However, most of the available data are from high-quality observational studies. Additional studies are necessary to improve the quality of evidence.

## Figures and Tables

**Table 1:** Primary studies included and their characteristics.

Author	Year	Sample	Patient	Image study	Index test	Outcome	Reference standard	Comments
Vilmann et al. [13]	2005	31	Lung cancer staging or suspected lung cancer	CT scan with suspected mass or lymph node	EBUS-TBNA + EUS-FNA	Lung cancer staging or diagnosis	Thoracotomy or clinical followup	Prospective trial, non-RCT. 9 patients underwent thoracotomy and 19 had clinical followup.

Wallace et al. [14]	2008	138	Lung cancer staging or suspected lung cancer	CT scan and PET CT with enlarged and/or PET positive lymph nodes	EBUS-TBNA + EUS-FNA	Lung cancer staging or diagnosis	Thoracotomy, mediastinoscopy, lobectomy, and thoracoscopy	Prospective trial, non-RCT. 33 patients underwent thoracotomy, 4 mediastinoscopy, 4 lobectomy, and 1 thoracoscopy. The rest had 6–12-month clinical followup.

Annema et al. [15]	2010	241	Lung cancer staging, resectable	CT scan and PET CT with enlarged and/or PET positive lymph nodes	EBUS-TBNA + EUS-FNA	Lung cancer staging	Mediastinoscopy and/or thoracotomy	RCT, 1 : 1. One arm to endoscopic staging and one arm to surgical staging. Standard reference for this study included thoracotomy in patients without positive endosonography.

Herth et al. [16]	2010	139	Lung cancer staging or suspected lung cancer	CT scan, PET CT in some patients	EBUS-TBNA and EUS-B-FNA	Lung cancer staging	Thoracoscopy, thoracotomy, or clinical followup to 12 months	Prospective study, non-RCT. Timing flow since inclusion is 6–12 months.

Hwangbo et al. [17]	2010	150	Lung cancer staging or suspected lung cancer	CT scan and PET CT with enlarged and/or PET positive lymph nodes	EBUS-TBNA and EUS-B-FNA	Lung cancer staging	Surgery, lymph node dissection	Prospective trial, non-RCT.

Szlubowski et al. [18]	2010	120	Lung cancer staging, stage IA-IIB	CT scan with normal size lymph nodes	EBUS-TBNA + EUS-FNA	Lung cancer staging	Bilateral transcervical extended mediastinal lymphadenectomy	Prospective trial, non-RCT. Patients with negative EBUS/EUS underwent bilateral transcervical extended mediastinal lymphadenectomy.

Ohnishi et al. [19]	2011	110	Staging for suspected resectable lung cancer	CT scan and PET CT with enlarged and/or PET positive lymph nodes	EBUS-TBNA + EUS-FNA	Lung cancer staging	Surgery without any specification	Prospective trial, non-RCT.

Szlubowski et al. [20]	2012	214	Lung cancer staging, stage 1A-IIIB	CT scan	EBUS-TBNA and EUS-B-FNA	Lung cancer staging	Systematic lymph node dissection	Prospective trial, non-RCT. 110 EBUS + EUS and 104 EBUS + EUS-B-FNA.

Kang et al. [21]	2014	148	Staging for confirmed or suspected resectable lung cancer	CT scan and PET CT with enlarged and/or PET positive lymph nodes	EBUS-TBNA and EUS-B-FNA	Lung cancer staging	Surgery without any specification	RCT, 1 : 1. EBUS centered arm versus EUS centered arm using the same bronchoscope. Patients without definitive data were excluded for sensitivity analysis.

Lee et al. [22]	2014	44	Staging for confirmed or suspected lung cancer	PET CT without M1 disease	EBUS-TBNA and EUS-B-FNA	Lung cancer staging	Mediastinoscopy or lymph node resection	Retrospective analysis. 4 patients underwent mediastinoscopy and 4 underwent lymph node resection.

Liberman et al. [23]	2014	144	Staging for confirmed or suspected resectable lung cancer	CT scan and PET CT with enlarged and/or PET positive lymph nodes	EBUS-TBNA + EUS-FNA	Lung cancer staging	Mediastinoscopy or lymph node dissection	Prospective trial, non-RCT. AS per protocol, patients underwent surgical staging following endosonographic staging.

Oki et al. [24]	2014	150	Staging for confirmed or suspected resectable lung cancer	CT scan and PET CT	EBUS-TBNA and EUS-B-FNA	Lung cancer staging	Surgical resection with lymph node dissection or clinical followup	Prospective trial, non-RCT. 5 patients were excluded from analysis without clinical followup. Clinical followup was 6 months after the procedure.

**Table 2:** EBUS + EUS adverse events reported in primary studies.

Author	EBUS + EUS adverse events
Vilmann et al. [13]	No complications
Wallace et al. [14]	No complications
Annema et al. [15]	One case of pneumothorax and 5 minor complications
Herth et al. [16]	No complications
Hwangbo et al. [17]	One case of lymph node abscess
Szlubowski et al. [18]	No complications
Ohnishi et al. [19]	No complications
Szlubowski et al. [20]	Two cases of nausea and 3 cases of self-limiting abdominal pain
Kang et al. [21]	12 cases of minor bleeding and 1 case of pneumomediastinum
Lee et al. [22]	No complications
Liberman et al. [23]	One case of bronchial laceration and 1 case of major bleeding
Oki et al. [24]	Two cases with severe cough

**Table 3:** Summary of meta-analysis of all included studies and subgroup analysis.

Comparison	Sensitivity	Specificity	LR (+)
All included studies	87.3% (CI 80–89%; *i* ^2^ = 22.86%)	99% (CI 99-100%; *i* ^2^ = 6.69%)	60.66 (CI 25.27–145.60; *i* ^2^ = 3.42%)
EBUS + EUS	85% (CI 80–89%; *i* ^2^ = 22.86%)	99.6% (CI 98.5–100%; *i* ^2^ = 6.69%)	60.66 (CI 25.27–145.6; *i* ^2^ = 3.42%)
EBUS + EUS-B-FNA	88% (CI 83.1–91.4%; *i* ^2^ = 25.64%)	100% (CI 99-100%; *i* ^2^ = 0%)	87.67 (CI 28.35–271.07; *i* ^2^ = 1.85%)

LR (+): positive likelihood ratio.

**Table:** (a) *EBUS + EUS pooled sensitivity*: 0.87 (95% CI: 0.83 to 0.89) | *pooled specificity*: 0.99 (95% CI: 0.99 to 1.00)

Test result	Number of results per 1000 patients tested (95% CI)	Number of participants (studies)	Quality of the evidence (GRADE)
	Prevalence 40.2%		
*True positives* (patients with staging)	350 (334 to 358)	609 (12)	*⨁⨁*◯◯ LOW^1,2^
*False negatives* (patients incorrectly classified as not having staging)	52 (68 to 44)		
*True negatives* (patients without staging)	592 (592 to 598)	906 (12)	*⨁⨁⨁*◯ MODERATE^3^
*False positives* (patients incorrectly classified as having staging)	6 (6 to 0)		

**Table:** (b) *EBUS-EUS-B-FNA pooled sensitivity*: 0.88 (95% CI: 0.83 to 0.91) | *pooled specificity*: 1.00 (95% CI: 0.99 to 1.00)

Test result	Number of results per 1000 patients tested (95% CI)	Number of participants (studies)	Quality of the evidence (GRADE)
	Prevalence 40.8%		
*True positives* (patients with staging)	359 (339 to 371)	297 (6)	*⨁⨁⨁*◯ LOW^1,2^
*False negatives* (patients incorrectly classified as not having staging)	49 (69 to 37)		
*True negatives* (patients without staging)	592 (586 to 592)	431 (6)	*⨁⨁⨁*◯ MODERATE^3^
*False positives* (patients incorrectly classified as having staging)	0 (6 to 0)		

^1^Low-quality studies.

^2^Imprecision between different studies

^3^Different standard reference.

**Table 5:** Excluded studies and motive for exclusion.

Author	Year	Motive for exclusion
Sánchez-Font et al. [25]	2014	Noninclusion criteria
Schuhmann et al. [26]	2014	Noninclusion criteria
Yarmus et al. [27]	2013	Noninclusion criteria
Yarmus et al. [28]	2013	Noninclusion criteria
Navani et al. [29]	2012	Noninclusion criteria
Fielding et al. [30]	2012	Noninclusion criteria
Oki et al. [31]	2012	Noninclusion criteria
Casal et al. [32]	2012	Noninclusion criteria
Steinfort et al. [33]	2011	Noninclusion criteria
Ishida et al. [34]	2011	Noninclusion criteria
Rintoul et al. [35]	2009	Noninclusion criteria
Chao et al. [36]	2009	Noninclusion criteria
Lee et al. [37]	2008	Noninclusion criteria
Yoshikawa et al. [38]	2007	Noninclusion criteria
Chung et al. [39]	2007	Noninclusion criteria
Yasufuku et al. [40]	2006	Noninclusion criteria
Herth et al. [41]	2006	Noninclusion criteria
Herth et al. [42]	2006	Noninclusion criteria
Herth et al. [43]	2003	Noninclusion criteria
Herth et al. [44]	2002	Noninclusion criteria
Verhagen et al. [45]	2013	Noninclusion criteria
